# Flotillins in Receptor Tyrosine Kinase Signaling and Cancer

**DOI:** 10.3390/cells3010129

**Published:** 2014-02-19

**Authors:** Antje Banning, Nina Kurrle, Melanie Meister, Ritva Tikkanen

**Affiliations:** Institute of Biochemistry, Medical faculty, University of Giessen, Friedrichstrasse 24, 35392 Giessen, Germany; E-Mails: Antje.Banning@biochemie.med.uni-giessen.de (A.B.); kurrle@med.uni-frankfurt.de (N.K.); Melanie.Meister@biochemie.med.uni-giessen.de (M.M.)

**Keywords:** cancer, receptor tyrosine kinases, flotillins, metastasis, mitogen activated proteins kinase, insulin, diabetes

## Abstract

Flotillins are highly conserved proteins that localize into specific cholesterol rich microdomains in cellular membranes. They have been shown to be associated with, for example, various signaling pathways, cell adhesion, membrane trafficking and axonal growth. Recent findings have revealed that flotillins are frequently overexpressed in various types of human cancers. We here review the suggested functions of flotillins during receptor tyrosine kinase signaling and in cancer. Although flotillins have been implicated as putative cancer therapy targets, we here show that great caution is required since flotillin ablation may result in effects that increase instead of decrease the activity of specific signaling pathways. On the other hand, as flotillin overexpression appears to be related with metastasis formation in certain cancers, we also discuss the implications of these findings for future therapy aspects.

## 1. Introduction

### 1.1. Receptor Tyrosine Kinase Signaling

This review summarizes the current knowledge on the role of flotillins in different signaling pathways that either start with the activation of receptor tyrosine kinases and/or culminate in mitogen activated protein kinase (MAPK) activation. For a review on flotillin functions in general, the reader is referred to two recent review articles [1,2].

The MAP kinase signaling cascade is the canonical downstream cascade of many growth factor receptors and plays a crucial role during carcinogenesis. In general, stimulation of receptor tyrosine kinases results in conformational changes of the receptor that allow autophosphorylation of tyrosine residues to take place. Subsequently, different signaling molecules are recruited to the activated receptors, which leads to activation of kinases such as the Ser/Thr kinase RAF (rapidly accelerating fibrosarcoma) which in turn phosphorylates and activates MEK (MAP kinase kinase, a dual specificity Tyr/Thr kinase) which is responsible for activating the MAP kinases such as extracellular signal regulated kinases 1 and 2 (ERK1/2). ERK1/2 themselves are promiscuous kinases and have almost 200 substrates that become phosphorylated upon activation of the MAP kinase pathway. These substrates can reside in various locations in the cell (e.g., cytoplasmic, nuclear). ERK1/2 signaling is tightly regulated and involves various feedback loops and phosphatases, *i.e.*, dual specificity phosphatases (DUSPs) that directly inhibit ERK1/2 activity (for a review see [3]). The output of ERK1/2 signaling depends on the substrates that are phosphorylated and can therefore be very diverse and include processes such as proliferation, differentiation or migration, but also survival or death. In general, deregulated ERK1/2 signaling is found in certain pathologies such as cancer [4]. Aberrant ERK1/2 signaling typically is the result of gain of function mutations of the small GTPase Ras [5] or the RAF kinase [6], both of which participate in ERK1/2 activation. In addition, activating mutations can also lie more upstream, and mutations of the EGFR occur frequently in several types of cancer (overview in [7]).

### 1.2. The Flotillin Protein Family

Flotillins, also called reggie proteins, form a family of two ubiquitously expressed and highly conserved proteins, *i.e.*, flotillin-1 and flotillin-2. Originally, they were discovered in goldfish as proteins whose expression is up-regulated in neurons after lesioning of the optic nerve. Because of their proposed function as neuronal regeneration molecules, they received the name “reggie” [8]. In the same year, flotillins were isolated from mouse lung tissue, and due to their ability to float in density gradients of Triton X-100 insoluble membrane preparations, they were named flotillin-1 and flotillin-2 [9]. Unfortunately, the numbering was opposite to the reggie nomenclature, with flotillin-1 being identical to reggie-2 and flotillin-2 to reggie-1. Meanwhile, the term “flotillin” has gained wider acceptance and has become the official gene name.

Despite being products of different genes, both flotillins have a molecular weight of approximately 48 kDa and their sequences are quite similar (50% identity on mRNA level and 44% on protein level). Flotillins are expressed in all mammals but they are also present in bacteria, plants, fungi and metazoans, but not e.g. in the budding yeast and *C. elegans* [10,11]. Flotillins are highly conserved among species. For example, mouse and human flotillin-1 share a 98.1% identical amino acid sequence [10]. Even between vertebrates and invertebrates the similarity is more than 60% [12], implicating that flotillins are important for cell physiology.

Officially, flotillins belong to the group of SPFH domain containing proteins [11,13]. This SPFH (stomatin/prohibitin/flotillin/HflK/C) domain and a C-terminal “flotillin domain” comprising alanine and glutamic acid repeats are the only recognizable domains within the flotillin protein. The SPFH domain, also referred to as prohibitin homology (PHB) domain, was first identified in stomatin [14] and is shared by several pro- and eukaryotic proteins. However, the function of the SPFH domain is still unknown. Most SPFH domain containing proteins tend to form oligomers, as was shown for stomatin [15,16], podocin [17], prohibitin [18] and flotillins, although the SPFH domain does not mediate the oligomerization of flotillins [19,20]. Another common feature of SPFH proteins is their association with lipid rafts (reviewed in [13]).

Typical with the SPFH domain containing proteins, flotillins also tend to form hetero- as well as homo-oligomers [19,20,21], but this oligomerization requires the flotillin domain [20,21] and tyrosine residue 163 of flotillin-2 [19]. Flotillins stabilize each other, which became obvious upon siRNA mediated knockdown of either flotillin-1 or -2 and concomitant decrease in the expression of the other one [20,22,23]. Notably, this interdependency is stronger in the case of flotillin-1, since flotillin-1 depletion typically results in only a moderate or even no depletion of flotillin-2, suggesting that flotillin-1 is more dependent on flotillin-2 than *vice versa*.

Flotillins were originally believed to be transmembrane proteins that are enriched in caveolae which are a subtype of membrane rafts [9,24]. However, this has been disputed in later studies where it was clearly shown that flotillins reside within non-caveolar rafts [25,26]. In general, membrane rafts are defined as dynamic, nanoscale membrane microdomains enriched in cholesterol and glycosphingolipids, which function as sorting platforms for various cellular processes [27]. Meanwhile, flotillins are widely used as membrane raft marker proteins. In contrast to what was assumed originally, they do not traverse the membrane, but are attached to the cytosolic leaflet of the plasma membrane by means of fatty acid modifications [21,28] and possibly hydrophobic stretches in the case of flotillin-1 [29]. Furthermore, flotillins harbor several putative, conserved phosphorylation sites [10,11], for some of which (*i.e.*, Tyr160 for flotillin-1, Tyr163 for flotillin-2) a functional role has been shown [30,31].

Flotillins are expressed ubiquitously, and their expression is particularly high in brain, heart, lung, and placenta, but fairly low in pancreas and liver [10,26]. Despite their ubiquitous tissue distribution, the expression of flotillins underlies, at least to some extent, transcriptional regulation and can be fine-tuned by different transcription factors [32]. Both flotillins are transcriptional targets of the MAPKs ERK1/2. The transcription factors Egr1 and serum response factor (SRF), both of which are ERK1/2 downstream targets, were identified as positive regulators of flotillin expression. Independently of MAPK activation, flotillin expression was shown to be induced by dimerization partners of the retinoid X receptor (RXR), such as PPARγ and RARα [32].

The subcellular distribution of flotillins is highly dynamic. Although they are present at the plasma membrane in many cell types, their localization is to some extent dependent on the cell type and on the differentiation status of the cell. In most cells, e.g., CHO, HepG2 and Hela cells, flotillins are predominantly found at the plasma membrane, while in MDCK cells they are mainly found in intracellular structures [29]. Flotillins have also been shown to be localized in endosomes and lysosomes [33], in exosomes (flotillin-1) [34,35], in the Golgi (flotillin-1) [36], and in the nucleus (flotillin-1) [37]. The expression of flotillins is often increased in differentiated cells, as has been shown for osteoclasts [38], skeletal myoblasts [24], and 3T3 fibroblasts [9].

## 2. Flotillins in Receptor Tyrosine Kinase and MAP Kinase Signaling

Many signaling pathways take place, at least partially, in membrane rafts. Flotillins as a major component of non-caveolar rafts have been suggested to participate in various signaling pathways involving receptors that are located in rafts. Due to their ability to form large oligomers, flotillins have been proposed to exhibit a scaffolding function in rafts. However, the fact that they become tyrosine-phosphorylated by Src family kinases upon growth factor treatment [30] and that they are able to associate with Src family kinases [33] also point to an active role of flotillins during signaling. Most studies have so far used an siRNA approach to deplete flotillin expression in cultured cells and analyzed the effects thereof on a given signaling pathway. Recently, also knockout mouse models for either flotillin-1 or flotillin-2 have been described [23,39,40]. We here summarize the results obtained using cultured cells and the data revealed by gene knockout studies in mice.

### 2.1. Cell Culture Studies

#### 2.1.1. Role of Flotillins in EGFR Signaling

The EGF receptor is a typical receptor tyrosine kinase and belongs to the ErbB family of receptors. After binding of a ligand, e.g., EGF, EGFR dimerizes and becomes autophosphorylated at several tyrosine residues. The activated receptor recruits different signaling molecules that facilitate the activation of downstream signaling pathways, in particular the MAPK pathway. Abnormally active EGFR signaling is typically found in many types of cancer (overview in [7]).

**Figure 1:**
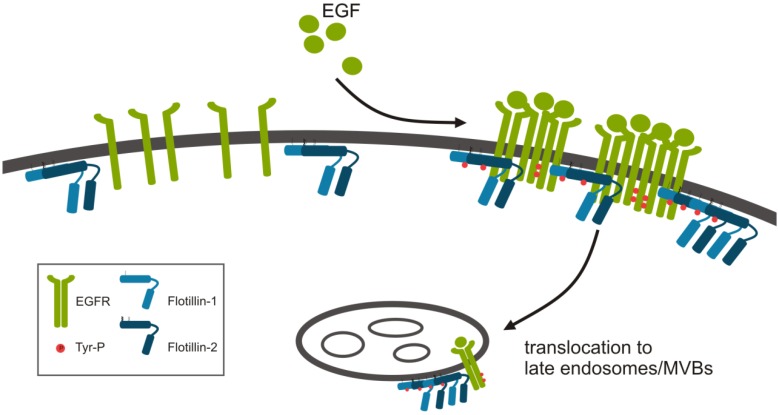
Modifications of flotillins upon epidermal growth factor (EGF) stimulation. Flotillins undergo modifications during RTK signaling. In addition to tyrosine phosphorylation upon EGF stimulation, flotillin hetero-oligomers increase in size and translocate to late endosomes.

Upon EGF stimulation, flotillins undergo several changes (Figure 1). Flotillin oligomers increase in size upon EGF stimulation [19]. EGF stimulation further leads to phosphorylation of flotillin-2 at several tyrosine residues by Src kinases [30] with whom flotillins have been shown to interact [30,41]. After growth factor stimulation, flotillins become endocytosed from the plasma membrane into late endosomes [30,31], and this EGF induced endocytosis requires the presence of Y160 in flotillin-1 [31] and Y163 in flotillin-2 [30,31]. The Src family kinase Fyn directly phosphorylates flotillins at Y160 (flotillin-1) and Y163 (flotillin-2), respectively [31]. However, if this tyrosine phosphorylation itself is a prerequisite for endocytosis is not clear yet. We have shown that EGF induced endocytosis of flotillin-2 also takes place upon inhibition of Src family kinases, but it requires hetero-oligomerization of flotillin-1 and -2 [19], while in another study inhibition of Src kinases prevented flotillin endocytosis [31]. Our data show that flotillins are not required for the endocytosis of EGFR itself [22]. However, a reduced EGFR internalization has been described in flotillin-2 deficient cells that overexpress very high amounts of EGFR [42].

**Figure 2:**
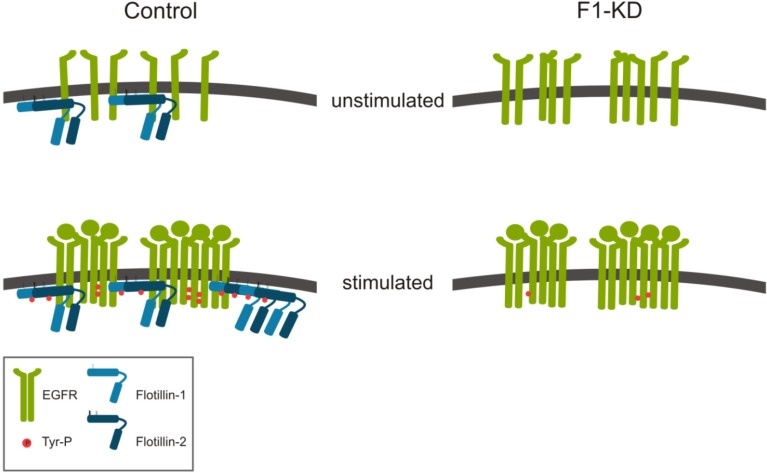
Flotillins in EGFR activation/clustering. While EGF stimulation of control cells induces EGFR activation, tyrosine phosphorylation and clustering of the receptor, cells depleted of flotillin-1 show a reduced tyrosine phosphorylation. Already in unstimulated cells, preformed EGFR clusters that do not increase in size upon EGF stimulation are observed in the absence of flotillin-1.

We have shown that flotillin-1 is required for a proper EGFR activation and downstream signaling. Flotillins form constitutive complexes with EGFR, and knockdown of flotillin-1 in HeLa cells results in a changed clustering behavior of EGFR at the plasma membrane upon EGF stimulation (Figure 2). In control cells, a portion of the activated cellular EGFR pool was recruited to membrane rafts, whereas in flotillin-1 knockdown cells, recruitment of EGFR and its signaling partners was reduced [22]. Furthermore, EGF-induced tyrosine phosphorylation of EGFR and activation of ERK1/2 was diminished in flotillin-1 knockdown cells [22]. However, flotillin-2 depletion did not display a similar effect in HeLa cells, whereas in A431 cells it also resulted in reduced EGFR phosphorylation [42]. We have shown that flotillins directly interact with several components of the MAPK cascade, *i.e.*, flotillin-1 forms a complex with cRAF, MEK1, ERK, and the kinase suppressor of RAS (KSR) [22]. Thus, flotillin-1 appears to function as a novel MAP kinase scaffolding protein (reviewed in [43]).

Surprisingly, flotillin-1 and -2 seem to have contrary effects on the activation of the MAPK ERK1/2. While the absence of flotillin-1 results in severe attenuation of the signaling cascade, most likely due to its function as a MAPK scaffolding protein, loss of flotillin-2 seems to have opposite effects. Knockdown of flotillin-2 in HeLa cells was accompanied by an increase in the basal phosphorylation of the cRAF kinase, MEK1/2 and ERK1/2, indicating that the ERK1/2 pathway is overactive in the absence of flotillin-2 [23]. In line with this, flotillin-2 knockdown cells showed an increased phosphorylation of MAPK substrates and also a higher proliferation rate. Furthermore, flotillin-2 knockdown cells express more Egr1 and Fos, both of which are typical ERK1/2 downstream targets. The increased expression of the transcription factor Egr1 resulted in a higher activity of its target gene p53, linking flotillins to cell survival and apoptosis [23]. Similar effects were also found in the flotillin-2 knockout mouse model [23], verifying the *in vivo* relevance of these findings (see Section 2.2).

The opposite effects of flotillin-1 and -2 on the activation state of ERK1/2 and the outcome of MAPK signaling is, at first glance, unexpected and surprising. However, it is not clear if the missing flotillin is directly causing this effect. It is possible that the remaining flotillin-2 in the flotillin-1 knockdown cells, which is now lacking its hetero-oligomerization partner, is forced to form only homo-oligomers that could be responsible for the observed effects. Furthermore, the outcome of flotillin depletion may also depend on the mode of knockdown (transient siRNA *vs.* stable shRNA/gene knockout) or the cellular background. We have recently shown that in MCF7 breast cancer cells, flotillin-1 depletion results in a paradoxical up-regulation of EGFR expression due to the increased phosphatidyl-inositol 3-kinase (PI3K) signaling ([44]; see Section 3 below). Thus, in order to elucidate the function of flotillins in signaling, cells or animals that completely lack flotillin-1 and -2 expression would be needed.

#### 2.1.2. Flotillins and FGFR Signaling

Fibroblast growth factors (FGF) bind to and activate the family of FGF receptors and thereby mediate their intracellular effects. The family of FGF receptors comprises 4 members (FGFR1-4), all of which are receptor tyrosine kinases. In response to FGF stimulation, the receptors become autophosphorylated and recruit different signaling molecules which then also become tyrosine phosphorylated (overview in [45]). Among those signaling molecules, the adaptor molecules fibroblast growth factor receptor substrate 2α (FRS2) and 2β (FRS3) are crucial for transmitting the signal farther downstream. FRS2 proteins bind to the FGFR with their phosphotyrosine binding domain (PTB domain) and become tyrosine phosphorylated upon FGF stimulation [46,47]. The binding to FGFR is constitutive and takes place in a region of FGFR that does not contain tyrosine residues [48]. Therefore, it is believed that this constitutively bound FRS2 is efficiently phosphorylated by the FGFR tyrosine kinase during receptor autophosphorylation [49]. Once FRS2 is tyrosine phosphorylated, it creates docking sites for SH2 domain containing proteins such as the adaptor molecule Grb2 or the protein tyrosine phosphatase Shp2, thereby activating the ERK1/2 pathway [47,50]. We have shown that both FRS2α and FRS2β are able to interact with flotillin-1 *in vitro* and also *in vivo* [51]. The interaction with flotillin-1 takes place at the PTB domain of FRS2α and facilitates the membrane association of FRS2α. Furthermore, it is required for the recruitment of FRS2α into membrane rafts. Consequently, in flotillin-1 knockdown cells, the FGF induced ERK1/2 phosphorylation is reduced. Due to the fact that flotillin-1 binds to the PTB domain of FRS2α, it competes for the binding with the FGF receptor. Hence, flotillin-1 is able to modulate the downstream signaling of FGF receptor, and the flotillin-1/FRS2 interaction is needed for proper MAPK activation upon FGFR signaling [51].

#### 2.1.3. Flotillins and Neurotrophin Receptor Signaling

The adaptor proteins FRS2α and FRS2β not only regulate the signaling downstream of the FGF receptor, but also of other receptor tyrosine kinases, such as the neurotrophin receptor TrkA that is activated upon nerve growth factor (NGF) binding. NGF promotes neuronal differentiation, a process that involves activation of ERK1/2. Similarly to FGF treatment, FRS2α and FRS2β are tyrosine phosphorylated upon NGF stimulation [46,47]. In contrast to the FGF receptors, binding of FRS2 to TrkA occurs at phosphorylated tyrosines and hence requires prior activation and tyrosine phosphorylation of the receptors [52]. TrkA signaling takes place in membrane rafts into which the activated receptor is recruited. This raft localization is required for optimal ERK1/2 activation and is mediated by the interaction of flotillin-1 and the adaptor protein c-Cbl associated protein (CAP). The interaction between flotillin-1 and CAP increases upon NGF treatment [53]. Flotillin-1 is known to specifically interact with the SoHo domain of CAP [54]. Mutation of the CAP SoHo domain inhibits the interaction with flotillins and the raft association of TrkA and results in attenuated ERK1/2 signaling. Most likely, by interacting with both flotillin-1 and TrkA, CAP serves as an adaptor to anchor TrkA to lipid rafts [53]. We have shown that both CAP and flotillin-1 can interact with the adaptor protein FRS2, and since they bind to overlapping sites, they appear to compete for the binding to FRS2 [51].

If flotillins also modulate the signal transduction of other receptor tyrosine kinases that utilize FRS2 and/or CAP as adaptor molecules is not known yet, with the only exception of the insulin receptor (see 2.1.4). Other candidate receptors whose interaction with FRS2 has been shown are RET, the receptor that transduces the signals of glial cell derived neurotrophic factors (GDNF) [55], and neuronal anaplastic lymphoma kinase (ALK) [56], both of which induce the activation of ERK1/2. Moreover, FRS2 is able to bind to the activated EGF receptor, and overexpression of FRS2 results in a dose dependent increase of EGF and FGF induced ERK1/2 activation [57]. Similar to the neurotrophin receptors, FRS2 binding to RET or ALK and its subsequent phosphorylation requires the receptors to be tyrosine phosphorylated [56,58]. FRS2 proteins also interfere with the vascular endothelial growth factor (VEGF) induced signal transduction that involves activation of the kinase insert domain containing receptor (KDR). FRS2 is constitutively associated with KDR, becomes tyrosine phosphorylated upon VEGF stimulation and recruits signaling molecules such as Grb2 [59].

As obvious from what we know about receptor tyrosine kinase signaling pathways that utilize the adaptor molecules FRS2α and FRS2β as docking sites for the recruitment of signaling molecules, FRS2 proteins play a pivotal role in coupling a subgroup of receptor tyrosine kinases with the MAPK signaling cascade. Due to the proven interaction between flotillin-1 and the PTB domain of FRS2 proteins and the requirement of flotillin-1 for proper membrane localization of FRS2 [51], a general role of flotillins in the fine tuning of FRS2 dependent signal transduction seems likely.

#### 2.1.4. Function of Flotillins in Insulin Signaling

Insulin is the primary hormone required for the maintenance of blood glucose levels. It leads to increased glucose uptake via the glucose transporter GLUT4 in skeletal muscle and adipose tissue, *de novo* synthesis of glycogen and inhibition of gluconeogenesis. Insulin signaling was originally believed to solely depend on the activation of PI3K. This canonical insulin signaling pathway comprises the following steps: After insulin binding, the insulin receptor is autophosphorylated and, hence, activated. After recruitment and tyrosine phosphorylation of insulin receptor substrates IRS-1 and -2, PI3K binds via its SH2 domain to the phosphorylated IRS and produces phosphatidylinositol (3,4,5)-trisphosphate (PIP3). PIP3 allosterically activates PDK which in turn phosphorylates Akt/PKB and the atypical PKCζ, leading to the translocation of GLUT4 from intracellular stores to the plasma membrane. In addition to this canonical insulin signaling pathway, an alternative pathway that is independent of PI3K but involves flotillin-1 and proceeds through membrane rafts has been described in adipocytes [60]. This alternative insulin signaling pathway, which most likely has a function in fine tuning the canonical insulin pathway, also starts with the activation and autophosphorylation of the insulin receptor. However, different signaling molecules, *i.e.*, CAP and c-Cbl, are now recruited to the receptor. CAP forms a complex with c-Cbl through one of its three SH3 domains. CAP also binds to the adaptor protein APS which in turn is bound to the activated insulin receptor [61] that phosphorylates c-Cbl [62]. The phosphorylated CAP/c-Cbl complex is then recruited to membrane rafts, and this recruitment requires the presence of flotillin-1 which directly interacts with CAP [60]. Within rafts, the phosphorylated c-Cbl recruits CrkII which binds to the GDP-GTP exchange factor C3G, leading to the release of GDP in exchange for GTP on the Rho family GTPase TC10. Activation of TC10 is required for GLUT4 translocation and takes place in rafts [63]. During insulin signaling, flotillins are not only responsible for the recruitment of CAP into membrane rafts, but also interact with GLUT4 and move together with GLUT4 from intracellular stores to the plasma membrane in adipocytes [64]. Interestingly, FRS2 is also phosphorylated upon insulin stimulation, and this phosphorylation is most likely carried out by the activated insulin receptor [65]. Since CAP and flotillin-1 interact with FRS2 in a competitive manner [51], it is plausible that this interaction might also be relevant for insulin signaling. However, this has not been directly investigated so far.

#### 2.1.5. Flotillins and Other Signaling Pathways

In addition to receptor tyrosine kinases, activation of MAPK can be induced by other signal transduction pathways, such as G protein coupled receptors (GPCRs). Heterotrimeric G proteins consist of α, β, and γ subunits. Depending on the α subunit, activation of GPCRs leads either to activation of phospholipase Cβ (Gα_q_), activation (Gα_s_) or inhibition (Gα_i/o_) of adenylate cyclase, or activation of Rho (Gα_12/13_). Gα_q_ has been shown to induce p38 [66,67] and Erk1/2 [66] MAPK activation. Flotillin-1 and -2 directly interact with Gα_q_, and knockdown of flotillin-2 led to attenuated p38 but not Erk1/2 MAPK activation upon treatment with UTP, an activator of the Gα_q_ coupled P2Y receptor [66]. The p38 activation was abolished upon Src kinase inhibition or cholesterol depletion, indicating that Gα_q_ mediated p38 activation requires active Src family kinases and cholesterol containing membrane microdomains. The interaction of flotillins with Gα_q_ required an intact N-terminal part of flotillin-1 and -2 and was independent of the nucleotide binding state of Gα_q_ [66]. Our unpublished data show that activation of ERK signaling in epithelial cells upon stimulation of the muscarinic acetylcholine receptors also requires flotillins. Therefore, flotillins most likely also fulfill a scaffolding function in the activation and signal transduction of G protein coupled receptors.

During the activation of the IgE receptor, FcεRI, which involves crosslinking of the receptor and recruitment into membrane rafts, the receptor becomes tyrosine phosphorylated by the Src kinase Lyn which is constitutively associated with flotillin-1. The activation of FcεRI is accompanied by a stimulation of the MAPK cascade. In flotillin-1 knockdown RBL-2H3 cells, the antigen induced phosphorylation of FcεRI and also of ERK1/2 was reduced. Most likely, flotillin-1 interferes with the kinase activity of Lyn and, hence, with the tyrosine phosphorylation of FcεRI, although the exact mechanism is not clear. Possibly, flotillin-1 might act as an adaptor protein that recruits another kinase which then phosphorylates and activates Lyn [41]. However, if flotillin-1 only acts at the level of receptor activation or functions again as a MAPK scaffolding protein, as we have shown for the EGFR signaling pathway [22], is not known yet.

### 2.2. Animal Models

#### 2.2.1. Flotillin-1 Knockout Mice

Flotillin-1 knockout mice are viable and fertile and do not show any obvious phenotype [40]. As expected from what we know from cell culture data, the expression of flotillin-2 is also reduced in these mice. The remaining flotillin-2 is not located in membrane rafts anymore, but shows a uniform distribution across the plasma membrane. Consistently, flotillin microdomains are absent in these mice. Normally, flotillin containing microdomains accumulate in stimulated, polarizing neutrophils in a plasma membrane protrusion referred to as the uropod [68,69,70]. In general, this uropod is important for proper leukocyte signal transduction, cell adhesion, migration, and apoptosis (for a review see [71]). In the case of the flotillin-1 knockout mice, neutrophil recruitment after treatment with the chemotactic bacterial peptide N-formyl-methionyl-leucyl-phenylalanine (fMLP) was reduced to 20%, which was explained by the requirement of flotillin containing microdomains for neutrophil recruitment and migration [40]. In addition, neutrophils isolated from flotillin-1 knockout mice show a reduced phosphorylation of the uropod component myosin IIa whose phosphorylation and hence activity is needed for uropod formation. From these data, it was concluded that flotillins spatially organize the membrane during neutrophil polarization and are therefore needed for proper uropod formation [40].

If the activation of receptor tyrosine kinases is affected in flotillin-1 knockout mice has not been analyzed so far. From what we know from cell culture studies [22], one would expect defects in EGFR as well as ERK1/2 activation. However, the activation of ERK1/2 in neutrophils upon fMLP treatment was not changed [40], but if this is also the case for other cell types and stimuli awaits further analysis.

#### 2.2.2. Flotillin-2 Knockout Mice

Flotillin-2 knockout mice were independently developed by two laboratories, including ours [23,39]. Similarly to the flotillin-1 knockout mice, they do not display any obvious phenotype or developmental defects. For the description of the mice generated by Berger *et al.* [39], please refer to Section 3.2. As expected, flotillin-2 knockout resulted also in a highly reduced expression of the flotillin-1 protein. The residual flotillin-1 was not located in membrane rafts anymore, but was found solely in high density membrane fractions, whereas caveolin-1 showed a normal distribution. This indicates that genetic ablation of flotillin-2 abolishes the formation of flotillin rafts but not that of the caveolae subtype of rafts [39]. Hence, the results from all flotillin knockout mouse models described so far emphasize that both flotillins are required for the formation of flotillin microdomains within membranes.

In order to analyze the global gene expression changes upon flotillin-2 knockout, we performed microarray experiments with RNA isolated from four different organs (liver, lung, kidney and colon). In all organs analyzed, flotillin-2 knockout animals displayed a significant up-regulation of ERK1/2 downstream effectors (e.g., Egr1, Fos) and their respective target genes, pointing towards an increased activity of the MAPK pathway in these mice. In addition, flotillin-2 knockout mouse embryonic fibroblasts (MEF) had a significantly increased basal ERK phosphorylation and activity which was accompanied by an increased phosphorylation of MAPK substrates [23]. These data are in line with the increased ERK1/2 activity that we have observed in cell culture studies where flotillin-2 was depleted by means of siRNAs. On the other hand, the dual specificity phosphatases Dusp1 and Dusp5, which are MAPK phosphatases and may thus negatively regulate MAP kinase signaling, also showed a higher expression in our flotillin-2 knockout mice and in flotillin-2 MEFs, most likely to compensate for the increased ERK1/2 activity [23].

## 3. Flotillins in Cancer and Metastasis Formation

Several recent publications have revealed that flotillins are associated with various types of human cancers. This has significantly advanced our understanding of the elusive cellular functions of flotillins. The largest amount of data has been obtained for melanoma and breast cancer which are reviewed below. Table 1 summarizes the literature on changes in flotillin expression in various types of human cancers.

**Table 1:** Changes in flotillin expression in human cancers.

Up-regulated	Cancer Type	Patient Phenotype	Ref.
**Flotillin-1**	Lung adeno-carcinoma	Expression correlates with advanced clinical stage, lymph node metastasis; decreased overall survival time	[72]
	Esophageal squamous cell carcinoma	Expression correlates with clinical stage and NF-κB activity; decreased overall survival time	[73]
	Breast cancer	Expression correlates with clinical stage and poor patient survival	[74]
	Hepatocellular carcinoma	Expression correlates with tumor size, clinical stage, CLIP stage, vascular invasion, relapse and serum AFP; decreased overall survival time	[72]
**Flotillin-2**	Gastric cancer	Expression correlates with histological type, Lauren grade, ErbB2 expression, lymphovascular invasion, lymph node metastasis and T-stage; decreased overall survival time	[75]
	Breast cancer	Expression correlates with clinical stage, T classification, M classification, histological differentiation, ErbB2 expression and poor patient survival	[39,76,77]
	Melanoma	Expression correlates with melanoma progression, lymph node metastases and Breslow depth	[78,79]

### 3.1. Flotillins in Breast Cancer

Breast cancer is the most common malignancy and the most deadly cancer among women world-wide, accounting for 23% of all new cancer cases and 14% of the total cancer deaths [80]. Up to date, most of the data functionally connecting flotillins to cancer were obtained in studies examining breast cancer or breast cancer cell lines. In line with this, the gene for flotillin-2 is located in a region on human chromosome 17q11.2 that is commonly amplified in human breast cancers [39]. One of the first functional links of flotillins to cancer was obtained in the study by Perou *et al.*, in which flotillin-2 was identified to be up-regulated in ErbB2 overexpressing, low-estrogen receptor (ER) expressing human breast tumors [81]. Later on, Lin *et al.* showed that the expression level of flotillin-1 significantly correlated with the clinical staging (stage I-IV) and poor breast cancer patient survival, and flotillin-1 depletion resulted in inhibition of proliferation and tumorigenicity of breast cancer cells *in vitro* and *in vivo* [74]. However, another study suggested that flotillin-2 but not flotillin-1 expression can be used as a prognostic marker for relapse in breast cancer in patients of stage I/II [76]. One reason for this discrepancy could be that different types of breast cancer samples were examined in regard to estrogen receptor and ErbB2 receptor expression, as several studies have provided evidence for a functional connection of flotillins with the ErbB family of receptors. Earlier studies have already shown that flotillin-2 is up-regulated in ErbB2 overexpressing breast tumor biopsies [81]. The ErbB2 gene is amplified or overexpressed to various degrees in distinct types of human cancers, with an approximate frequency of 20%–30% in breast cancer [82,83,84]. The receptor tyrosine kinase ErbB2, also known as human epidermal growth factor receptor 2 (Her2), is a member of the epidermal growth factor receptor family (EGFR/ErbB family), which in humans also comprises the EGFR, Her3/ErbB3 and Her4/ErbB4 (reviewed in [85,86]). Overexpression of flotillin-2 in breast cancer was shown to be associated with the clinical stage, T and M classification, histological differentiation and receptor tyrosine kinase ErbB2 expression levels [77]. Furthermore, the same study demonstrated that flotillin-2 is up-regulated on protein and mRNA level in various breast cancer cell lines as well as in breast cancer tissues in comparison with adjacent non-cancerous tissue samples. Similar results were also obtained with gastric cancer tissue biopsies in which flotillin-2 expression positively correlated with ErbB2 expression [75]. In an ErbB2 overexpressing breast cancer cell line, SKBR3, flotillin-1 and flotillin-2 partially colocalized with ErbB2 at the plasma membrane and formed a complex with ErbB2 and Hsp90 [76] which is an important factor for the stabilization of ErbB2 at the plasma membrane [87]. On the other hand, Hsp90 has also been shown to regulate ErbB2 signaling by restricting its heterodimerization with other ErbB family members [88]. Transient depletion of either flotillin-1 or flotillin-2 leads to an increased internalization of ErbB2, implicating that flotillins play a role in ErbB2 stabilization at the plasma membrane [76]. However, the observed effect on ErbB2 was more pronounced upon depletion of flotillin-2, which in SKBR3 cells also results in a profound loss of flotillin-1. Pust *et al.* also demonstrated that the phosphorylation of ErbB2 at tyrosine 1248 was significantly reduced after flotillin-1 or flotillin-2 depletion. However, knockdown of flotillin-2 but not of flotillin-1 decreased the phosphorylation of protein kinase B/Akt [76], indicating that different signaling modes may be differentially dependent on flotillins. These data are contradictory to the findings of Lin *et al.* who showed a significantly decreased phosphorylation of Akt upon flotillin-1 depletion in the breast cancer cell lines MCF7 and MDA-MB-231 [74]. It is not completely clear what causes these differences but one reason might be the status of ErbB2 expression of the cell lines used, since MCF7 and MDA-MB-231 cells do not overexpress ErbB2 [89], whereas SKBR3 cells do. Another reason for the varying results might be the method used for flotillin depletion, *i.e.*, transient (siRNA) or stable (shRNA). We have recently shown that in MCF7 cells which express very low levels of EGFR [89], a stable shRNA mediated knockdown of flotillin-1 resulted in a significant up-regulation of EGFR, whereas no changes in protein expression of the related proteins Her2/ErbB2 and Her3/ErbB3 were detected [44]. The up-regulation of EGFR directly correlated with the loss of flotillin-1 expression, since in flotillin-2 depleted MCF7 cells, which express about 40% flotillin-1, a less pronounced up-regulation of EGFR was observed. Consistently, up-regulation of EGFR in flotillin-1 depleted MCF7 cells resulted in the activation of Akt and the MAP kinase pathway upon EGF stimulation. Since our earlier data show that in other cell types, flotillin-1 depletion rather causes a decrease in EGFR and MAPK activation [22], it is likely that the up-regulation in MCF7 cells represents a compensatory mechanism, similar to the one observed in our flotillin-2 knockout mice [23]. Intriguingly, we were able to show that inhibition of PI3K in flotillin-1 depleted MCF7 cells resulted in a reduction of EGFR expression [44]. Since MCF7 cells exhibit a constitutively active, E545K mutated form of the catalytic subunit of PI3K [90], the genetic background of the cancer cells used may also affect the effects seen upon flotillin depletion. Therefore, further studies are required to elucidate the precise function of flotillins in breast cancer in regard to the expression of the members of the EGFR family.

### 3.2. Flotillins in Melanoma and Metastasis Formation

The first direct demonstration of functional association of flotillins with cancer were the studies by Hazarika *et al.* who observed an increased flotillin-2 expression upon melanoma progression in various tumorigenic and metastatic melanoma cell lines *in vitro*, as well as in melanocytic lesions *in vivo* [78]. Overexpression of flotillin-2 in the human non-tumorigenic and non-metastatic cell line SB2, derived from a primary cutaneous melanoma [91], induced tumorigenicity and metastases. Furthermore, these cells showed an increased level of the GPCR protease activated receptor 1 (PAR-1) whose expression has been shown to be associated with metastatic potential in prostate cancer and in melanoma cells [92,93]. The same group also showed that flotillin-2 expression is associated with Breslow depth and lymph node metastasis in melanoma [79]. These studies strongly suggested that flotillin-2 might be involved in the process of metastasis formation, which marks the turning point from a local disease to a systemic, non-curable situation, thus having an extraordinary impact on the patients’ prognosis. In addition, these observations point to a functional role for flotillins in migratory processes, which is well in line with previous studies in cell culture systems and in the flotillin-1 knockout mouse. We have shown that a transient knockdown of flotillin-2 in HeLa cells impairs cell spreading, whereas overexpression of flotillin-2 enhances spreading and induces filopodia like protrusions in an expression level dependent manner in several epithelial cells lines [21,30]. Furthermore, studies on neutrophils have strongly suggested a functional role of flotillin microdomains during neutrophil migration [40,68,69,70,94].

Melanoma is not the only type of cancer in which flotillins have been associated with metastasis formation. Zhang *et al.* recently demonstrated that flotillin-1 protein expression is strongly increased in tissue samples obtained from lung adenocarcinoma in comparison to paraneoplastic normal lung tissue. In addition, it was shown that flotillin-1 expression increased with the advanced clinical stage and the development of lymph node metastasis. Also, in hepatocellular carcinoma and esophageal squamous cell carcinoma cells and patient samples, expression of flotillin-1 was shown to be increased and positively correlated with disease stage [72,73]. Furthermore, elevated flotillin-1 expression levels correlated with decreased overall survival time of the patients. Interestingly, the study by Song *et al.* for the first time demonstrated that flotillin-1 activates tumor necrosis factor-α receptor (TNFR) signaling and sustains activation of NF-κB [73].

The strongest argument for a specific role for flotillins in metastasis formation has been provided by Berger *et al.* who bred their flotillin-2 knockout mice with the MMTV-PyMT (mouse mammary tumor virus-polyoma middle T antigen) mice, which is an established transgenic mouse model for studying breast cancer. Very intriguingly, they showed that the formation of macro and micro lung metastases was significantly reduced in the mice lacking flotillin expression, whereas there was no significant effect on primary breast tumors [39]. Very recent findings have also shown that flotillin-1 is a target of the micro-RNA *miR-124* which regulates its translation. Interestingly, *miR-124* is frequently down-regulated in breast tumors, which correlates with an increased flotillin-1 expression [95]. Since *miR-124* down-regulation is also associated with metastasis formation and cell migration, some of these effects might be caused by the concomitant flotillin-1 up-regulation upon loss of *miR-124*. Taken together, these findings suggest that flotillins might be useful as potential biomarkers for metastasis formation and thus also for patient prognosis in various types of cancers.

## 4. Conclusions and Future Perspectives

A plethora of studies have revealed a vital role for flotillins in the regulation of cellular signaling, survival and proliferation. Numerous studies have also shown that overexpression of flotillins is a feature exhibited by several types of cancers and in many cases correlates with the disease stage, prognosis and patient survival. This has raised interest in the possibility that flotillins might be good targets for cancer therapy approaches. Since genetic ablation of flotillins is well tolerated in the knockout mouse models, this appears at first glance to be a promising future approach and would be expected to result in impairment of cancer cell proliferation and viability. However, our recent data have revealed that both stable depletion of flotillins in cell culture systems and their genetic ablation in the mouse may have quite a contrary effect to the one expected [23,44]. Some signaling pathways may become hyper-activated and signaling proteins overexpressed upon loss of flotillin expression, which most likely represents a compensatory mechanism. Our data revealed that the EGFR overexpression observed upon flotillin-1 depletion in breast cancer cells was due to the constitutively active PI3 kinase present in these cells [44]. Therefore, our findings call for great care when designing cancer therapy approaches targeting flotillins and show that it is important to know the molecular signature of the cancer cells, since specific mutations in other cancer relevant genes may render flotillin based approaches unsuitable for therapy.

Another interesting point that emerges from the studies addressing flotillins in cancer is that they may be specifically associated with the formation of metastases, at least in some cancer types. As only a limited number of genes have been described that show a specific function during metastasis formation, this may open up novel possibilities for a specific treatment of cancers such as melanoma, in which metastasis formation displays a major problem for the survival of the patient. Thus, therapies that target flotillins in cancer should be developed as they may well be suitable for a subset of up to date highly lethal cancers, as long as the possible adverse effects are kept in mind and carefully controlled.
